# ‘Let's Just Hope for the Best’: A Photovoice Study Exploring the Impact of Covid‐19 Restrictions on Children With Intellectual Disabilities in the UK

**DOI:** 10.1111/jar.70298

**Published:** 2026-08-28

**Authors:** Ally Pax Arcari Mair, Grace Khawam, Hope Christie, Karen Goodall, Doug McConachie, Jo Van Herwegen, Joanna Moss, Carrie Ballantyne, Hayley Crawford, Caroline Richards, Thomas Gallagher‐Mitchell, Laura Outhwaite, Karri Gillespie‐Smith

**Affiliations:** ^1^ Department of Health and Clinical Psychology, School of Health in Social Science University of Edinburgh Edinburgh UK; ^2^ Institute of Education University College London London UK; ^3^ School of Psychology University of Surrey Surrey UK; ^4^ Division of Psychology School of Education and Social Science, University of West of Scotland Scotland UK; ^5^ Division of Health Sciences, Warwick Medical School Warwick University Warwick UK; ^6^ School of Psychology University of Birmingham Birmingham Alabama USA; ^7^ Department of Psychology Liverpool Hope University Liverpool UK

**Keywords:** children, coping, Covid‐19, intellectual disabilities, mental health, photovoice

## Abstract

**Background:**

To date, little research has explored children with intellectual disabilities' own experience of the Covid‐19 pandemic. The current study used photovoice to explore the impact of Covid‐19 on children with intellectual disabilities.

**Method:**

Sessions were carried out with (*n* = 13) children aged 8–16 years across five sessions with five small groups (either face‐to‐face or video calls) between November 2021—March 2022.

**Results:**

Three main themes (1) “Nobody likes the lockdown”; (2) “You don't want the Covid [to] get you”; (3) *Everything is Different Now* were connected to an overarching theme of *(Im)Permanence*.

**Conclusions:**

Children with intellectual disabilities were anxious during the Covid‐19 pandemic and employed numerous coping strategies (such as distracting themselves with sensory‐based activities). Despite looking forward to seeing family/friends, children reported worries and hesitancy about returning to ‘normal’, indicating potential lasting effects of the lockdown.

## Lay Summary

- The current study is the only study to date that directly asks children with Intellectual Disabilities about their experiences during Covid‐19 Lockdown.
- Through Photovoice methods, children with intellectual disabilities talked about their experiences of the Covid‐19 Lockdown and mentioned how they didn't like the lockdown, that they were scared about covid ‘getting them’ and how everything now seems different.
- Children were anxious during the pandemic and employed numerous coping strategies, such as doing activities related to their favourite interests and doing sensory based activities (e.g., rituals using hand sanitiser).
- This research highlights that children with intellectual disabilities did have high anxiety during the pandemic and this may continue to affect them as they try to return to routines that existed pre‐Covid.

## 1 Introduction

Parents of children with intellectual disabilities faced additional barriers to everyday life during Covid‐19 lockdown due to difficulty accessing adequate support, and reported higher rates of stress than families with children without intellectual disabilities (Gillespie‐Smith et al. 2021; Sideropoulos et al. 2021). Despite research reporting how parents of children with intellectual disabilities were impacted during the pandemic (Gillespie‐Smith et al. 2021; Gillespie‐Smith et al. 2024; Sideropoulos et al. 2021), to the author's knowledge there has been no research documenting the experience of the pandemic from children with intellectual disabilities themselves. Despite the covid lockdown taking place 3 years ago, the effects are still being experienced to this day especially in children with intellectual disabilities evidenced by increased levels of anxiety (McDonald et al. 2023), and high levels of school absenteeism (Totsika et al. 2024). Therefore, there is a need to understand and obtain first‐hand accounts of how the Covid‐19 lockdown impacted these children.

### 1.1 Impact of Covid‐19 on Children

It is documented that Covid‐19 had a substantial impact even on neurotypical children. O'Sullivan et al. (2021) interviewed children, adolescents (*n* = 45) and their parents (*n* = 49) about the impact of Covid 19. Parents reported that the children struggled with mental health due to missed milestones, and that they were often ‘anxious and teary’. Children talked about the difficulties doing school work at home and how online learning was overwhelming and difficult to manage. Montreuil et al. (2023) interviewed children and adolescents (*n* = 25) and found similar results where children reported their poor mental health was caused by loss of social activities, stress of online learning and the imposed pandemic rules. The children also talked about the increased family time in close proximity, which helped, deepen close bonds but also increased rates of arguments and irritation. Another study by Larivière‐Bastien et al. (2022) focused on experiences of socialising and friendships during Covid‐19 specifically and interviewed children and adolescents (*n* = 67). Interestingly the children talked about how school was not only an important part of their lives but was central to their abilities to socialise. The children noted that they missed attending school, due to the diverse opportunities to spend time with friends that school provides.

### 1.2 Impact of Covid‐19 on Adults With Intellectual Disabilities

Despite there being a lack of qualitative research, capturing the experience of Children with Intellectual Disabilities there is research capturing adult experiences. One study by Lake et al. (2021) explored the mental health and wellbeing of adults with Intellectual Disabilities (*n* = 9) during Covid‐19 using interviews. They found that the pandemic impacted the participant's daily life and wellbeing with many of the adults reporting a rise in mental health issues (such as anxiety and depression) as well as an increased fear of death. Similar findings were reported in a study by Kim et al. (2021) who carried out research in Adults with Intellectual Disabilities. They further mentioned the impact of lockdown on family relationships, with the participants reporting that despite contributing to the household more (to reduce perceived caregiver burden) family conflict still increased, exasperating their mental health. Similar findings were reported in a study of college students with Intellectual Disabilities by Carey et al. (2021). Their participants noted increased mental health issues, responsibilities and anxiety caused by uncertainty about the future. These qualitative findings are supported by survey‐based studies which show increased mental health issues and lower quality of life for adults with Intellectual Disabilities during Covid‐19 pandemic (e.g., Amor et al. 2021; Rosencrans et al. 2021; for a scoping review see Keenan and Doody 2023).

### 1.3 Coping and Mental Health

Studies outlined above also mentioned how children coped during covid including talking to friends and family online (Larivière‐Bastien et al. 2022), dancing, art, video games and spending time with pets as well as having open communication with their parents (Montreuil et al. 2023). Similarly, adults with Intellectual Disabilities coped by doing the same type of activities (Kim et al. 2021), particularly noting meeting with friends and family virtually to reduce feelings of loneliness and isolation (Lake et al. 2021). Coping strategies are important to consider since research has shown that coping can lead to anxiety and depression (Fluharty et al. 2021) as well as psychological distress (Gillespie‐Smith et al. 2021; Gillespie‐Smith et al. 2024). These studies show a direct link between coping strategies and mental health during the pandemic in UK parents of children with Intellectual Disabilities; however, little is known about coping strategies employed by children with intellectual disabilities themselves. To the authors' knowledge there has been no research exploring coping strategies in children with intellectual disabilities by asking the children themselves.^1^ This is interesting given that survey‐based research reports 40% of children and adolescents with an intellectual disability are at increased risk of mental health problems, such as depression and anxiety (especially around specific phobias) when compared to neurotypical children (Totsika et al. 2022).

### 1.4 Photovoice

The limited first‐hand research carried out looking at mental health in children with intellectual disabilities is perhaps due to a lack of research methods that can be truly accessible and inclusive. Photovoice methodology is an inclusive method that enables experiences to be collected through images, which are then used as a basis for reflection and discussion (Rose 2014). Photovoice is a method where participants (i.e., community members often under‐represented in research) are positioned as experts in understanding their experiences (Wang and Burris 1997), enabling them to be empowered and actively involved in the research process (Wass and Safari 2020). Photovoice also allows opportunities for people (who need support with verbal communication) to self‐represent their experiences using artwork or images (Booth and Booth 2003; Williamson et al. 2020). Despite ethical concerns around taking photographs and children with intellectual disabilities having limitations in memory, attention and expressive language (Chinn and Balota 2023), photovoice is therefore a method that can provide them with a voice (Overmars‐Marx et al. 2018) which can lead to positive outcomes such as a feelings of achievement (Akkerman et al. 2014). The photovoice method still requires a level of understanding so although it is more inclusive than other methods—it still cannot be used by those who have severe and profound intellectual disabilities. Despite this, a recent systematic review shows photovoice methods have been successfully used in heterogeneous participant groups who have intellectual disabilities (for a review see Chinn and Balota 2023).

### 1.5 Current Study

The current study aimed to use photovoice methods to capture the perspectives and experiences of children with intellectual disabilities during Covid‐19 lockdown. The research question is ‘What is your experience of Covid‐19 and the Covid‐19 lockdown(s)?’

## 2 Method

### 2.1 Participants

Participants were children who had mild or moderate intellectual disabilities (*n* = 13) aged 7 to 18 years old (7 M: 6F) and were recruited via charities across the UK. Children could take part if they could understand simple instructions and information in English. Children were excluded if they had other disabilities but not Intellectual Disability and/or severe or profound Intellectual Disabilities. Photovoice methods were carried out across five online sessions with five small groups. All groups took part in the study online except for Group 5, which was conducted face‐to‐face. The photovoice sessions took place between November 2021 and March 2022. Participants represented a range of diagnoses, which included Mild/Moderate Intellectual Disability, Down Syndrome, Williams Syndrome, and Autism with Intellectual Disability (see Table 1). Groups were formed based on whether the children were at primary school or secondary school. Therefore, there was one group of primary school aged children (*n* = 2) and four groups of secondary school aged children (*n* = 2–4).

**TABLE 1:** Participant information.

Name	Sex	Age	Diagnoses	Photovoice group
Simon	M	18	Mild/Moderate ID + Epilepsy	Group 1
Meera	F	16	Down Syndrome	Group 1
Matthew	M	15	Down Syndrome	Group 1
Katie	F	17	Down Syndrome	Group 2
Hannah	F	13	Down Syndrome	Group 2
Rebecca	F	17	Williams Syndrome	Group 3
Maya	F	16	Autism + Williams Syndrome	Group 3
Ahmed	M	7	Down Syndrome	Group 4
Omar	M	7	Mild/Moderate ID + Autism	Group 4
Sarah	F	18	Mild/Moderate ID	Group 5
Fergus	M	17	Mild/Moderate ID	Group 5
Ryan	M	17	Mild/Moderate ID	Group 5
Mark	M	18	Mild/Moderate ID + Autism	Group 5

*Note:* All names are pseudonyms.

Abbreviations: F = Female; ID = Intellectual Disability; M = Male.

### 2.2 Procedure

Following ethical approval from the Department of Clinical and Health Psychology at the University of Edinburgh (CLPS099), the study was advertised on UK charity websites, and social media pages. A full protocol was written up and pre‐published which provides a detailed description of all the sessions (please see https://osf.io/8pcsm). Parents who were interested in their children taking part in the study clicked a link on the advert, which took them to the participant information sheet, consent form, basic demographic questions and contact details (hosted on Qualtrics). The study employed multimodal visual methods across 5 sessions (based on Amos and Lordly 2014) and were carried out by two researchers (GK and HC). The sessions were broken down as—Session 1—Introductory session for assent and trust‐building; Session 2—Storytelling (to gather retrospective information); Session 3—Camera training workshop and first photo assignment; Session 4—Discussion on the first assignment and second photo assignment; Session 5—Discussion on all photographs and wrap up. For more detailed information on the content of each session please see Supporting Information. Sometimes sessions were adapted for online participation by combining two sessions into one (for e.g., session one was combined with session two) to help sustain attention and motivation. Each online session lasted between 60 and 90 min. The children were asked to take photographs of what they thought was important about the way Covid‐19 has impacted their lives using their own smartphones and tablets. The researcher also allowed the children to practice taking a picture in the middle of a session and bring it back to the group for discussion. This showed the children that they were the key decision makers and were able to take images of anything they thought was important, ensuring they were empowered and understood their own agency. This practice also allowed the researcher to assess the level of child's engagement and to provide feedback on the process. When children brought pictures for discussion during the sessions they were encouraged to highlight the most important pictures, explain their meaning and also assign a title to these images.

**FIGURE 1:**
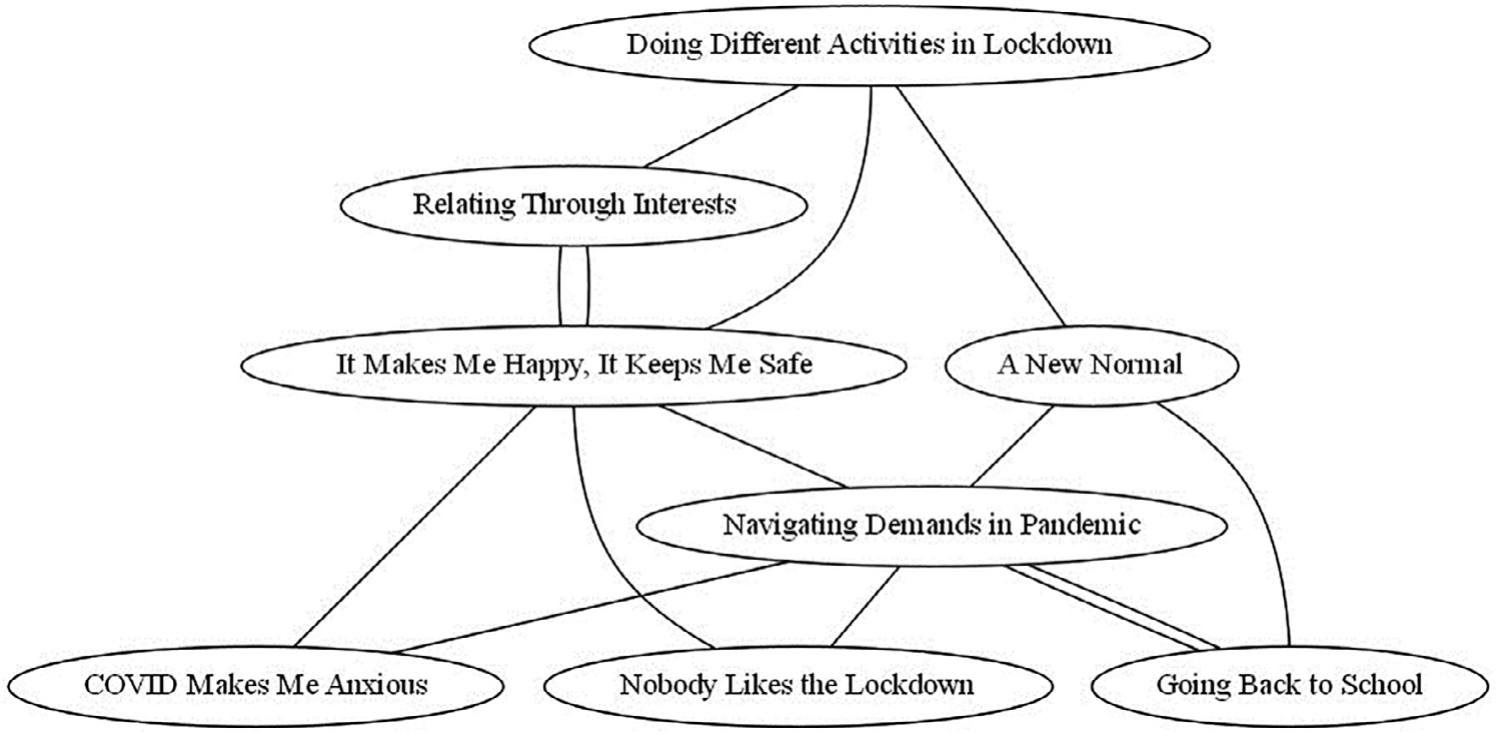
Developed thematic map.

The analysis framework for the study was adapted from Wang and Hannes' (2020) Photovoice Question Matrix and was employed by the researchers, looking at both the drawings and photographs, to assess the production of the image, the content of the image, and the meanings attributed to the image, and how this relates to the child's lived experience. Content of the images and transcripts was coded, and themes were established using inductive reflexive thematic analysis (Braun and Clarke 2019; 2021). NVivo was used to code transcripts and subsequently form aggregate nodes, which were used to identify themes and subthemes in the data. Themes from the images and transcripts were then further synthesised using triangulation (Tracy 2010) to identify textual and image themes. Theme reports were produced, and a developed thematic map was drawn using PyDot (see Figure 1), which highlighted connections between themes, further refining themes and collapsing themes where appropriate. Themes were then checked by the first author, and following discussions, refined themes were then shared with the wider research team.

Final themes and sub‐themes were identified, and the researcher reviewed the transcripts to ensure that the themes identified reflected the participants' own narrative(s). The images and themes were collated and a summary was sent to the parents to share with their children. Images and themes were also presented to stakeholder groups including parents of children with intellectual disabilities, charities, teachers and policy makers followed by a discussion. The discussion was transcribed and thematically analysed to form part of a parliamentary report submitted to the UK parliament (see https://www.ed.ac.uk/files/atoms/files/road_to_recovery_‐_roundtable_report_nov21.pdf).

### 2.3 Reflexivity

A constructive reflexive thematic approach was taken which views that in addition to the recurrence of important information, meaningfulness drives the development and interpretation of codes and themes (Byrne 2022). In engaging in critical reflexivity, it is important to acknowledge not only one's own positionality, but the relationships and connections, which build the context of that positionality. In this sense, it is acknowledged that the researcher who conducted the focus groups and the researcher who conducted the analysis, although sharing similar experiences of neurodivergence with the participants (the researcher collecting the data is a parent of a child who has intellectual disabilities and the researcher who conducted the analyses is autistic with significant communication difficulties), the researchers can only ever know the participants' experience in partiality, as they do not have an intellectual disability themselves and were also not a child during lockdown. Additionally, when considering other forms of intersectionality which cause our identities to be simultaneous, inter‐related and sometimes contradictory (Muhammad et al. 2015); it is important to acknowledge how being both an adult and researcher may have influenced perceptions of power throughout the study. The researchers attempted to mitigate this as much as possible (by building rapport, talking about the children's interests as well as encouraging the children's own agency in image taking, assigning titles and meaning‐making). In addition, the researcher who carried out the analysis sought to (re)present the participants' narratives, perspectives, and understanding in the participants' own words and images. Members of the wider team (who were less involved in the data collection and analyses) consisted of parents who experienced lockdown with their own children and have themselves spent years working with young children who have intellectual disabilities.

Reflexive journalling, as recommended by Braun and Clarke (2006), was conducted throughout the analysis; however, as Kohl and McCutcheon (2015) highlight, it is not simply enough to engage in individual practices of self‐reflexivity, rather one must embody these practices through *everyday talk* to examine their positionalities. This was established through appropriate *kitchen table reflexivity* with the researcher's peers insofar as to better understand the researcher's own positionality and any countertransference, which occurred whilst working with the data (Hollway, 2016).

## 3 Results

Three main themes with associated sub‐themes were identified from the analysis, which are connected to an overarching theme of *(Im)Permanence*: (1) “Nobody likes the lockdown”; (2) “You don't want the Covid [to] get you”; (3) *Everything is Different Now* (see Tables 2 and 3).

**TABLE 2:** Themes from thematic analysis.

Overarching theme	Theme	Sub‐theme
(Im)permanence	1. “Nobody likes the lockdown”	1a. “Stuck Inside”
		1b. “COVID makes me anxious”
		1c. “I just want COVID to leave”
	2. “You don't want the COVID [to] get you”	2a. Obeying Lockdown Rules
		2b. Sense of Control
		2c. It keeps me safe
	3. Everything is Different Now	3a. “We stopped going”
		3b. “It made me happy”
		3c. A New Normal
		3d. Back to School

**TABLE 3:** Brief overview of themes and descriptions.

Theme and Sub‐themes	Description
Overarching Theme: (Im)Permanence	The overarching theme of *(Im)permanence* is understood as the state, understanding, and desire of permanence in conflict with the notion of impermanence associated with the COVID‐19 pandemic and lockdown. This was often discussed throughout the three main themes, as change and routine being in tension with one another.
Theme 1: “Nobody likes the lockdown”	Theme 1 highlights the frustration and stress participants observed as a result of the pandemic and lockdown(s), and the impact this has had on their lives after lockdown.
Theme 1a: “*Stuck Inside”*	“*Stuck Inside*” reflects participants experiences of stress, frustration, and anxiety in lockdown due to the sense of containment they observed.
Theme 1b: “*COVID makes me anxious*”	Theme 1b highlights the impact lockdown and the pandemic had on participants' understanding of mortality and sense of wellbeing.
Theme 1c: “*I just want COVID to leave*”	Participants generally expressed a strong desire for “*COVID to leave*” and a return to a perceived normality. However, although the wish for COVID‐19 itself to leave was a factor, participants were generally more concerned with change and a wish for the new rules and routines of the pandemic to be replaced with a greater sense of normality and permanence that the participants observed prior to lockdown.
Theme 2: “*You don't want the COVID [to] get you*”	Theme 2 covers the stress and anxiety, which participants attributed to the new rules and changes to everyday life imposed by lockdown(s), the need participants felt to establish some sense of control and permanence, and the coping mechanisms they employed to feel a greater sense of safety.
Theme 2a: Obeying Lockdown Rules	Participants observed many new and competing demands emerging from the pandemic and the lockdown(s) they experienced. Although participants sought to understand the reasoning behind these new rules, they generally felt increasingly stressed in performing them.
Theme 2b: Sense of Control	Participants often sought fun and creative ways in which they could feel more in control of their lives during the pandemic, using their specific interests.
Theme 2c: It keeps me safe.	Participants often employed different tactics to feel a greater sense of control in order to feel safe. These included the use of specific interests, landmarks, sensory experiences, and spirituality, and also included more distressing cycles of rumination and compulsions.
Theme 3: Everything is Different Now	Theme 3 discusses the losses and gains participants noted in their day‐to‐day lives during and after lockdown.
Theme 3a: “*We stopped going*”	Participants observed a sense of grief over the loss of specific activities and routines in the transition in and out of lockdown(s); these were noted in the loss of specific social connections and activities as a result of being in a “*big old bubble*” and the uncertainty participants felt over a future of a world.
Theme 3b: “*It made me happy*”	Participants typically found the new activities offered during lockdown made them happy, however, the key aspect which appeared to bring the most joy to participants during lockdown was simply being able to spend more time with family and do more activities they enjoy with their family.
Theme 3c: A New Normal	Participants discussed a new normal they felt they entered into, the lack of certainty and permanence these transitions seemed to bring, and their excitement for a return to a perceived sense of normality whilst also grieving the loss of connections and activities which brought them joy and comfort during lockdown.
Theme 3d: Back to School	Participants observed a longing to return to in‐person education, yet typically found difficulty in a transition back into in‐person schooling as they felt they were no longer “*used to it*”; nevertheless, the participants highlighted their enjoyment in returning to in person schooling and seeing their friends again.

### 3.1 Overarching Theme: (Im)permanence

The overarching theme of *(Im)permanence* is understood as the state, understanding, and desire for permanence, which is in conflict with the notion of impermanence. This was often discussed throughout the three main themes as change and routine being in tension with one another:

**Hannah**: [How I felt about Covid‐19 was] not angry, but it's just happy but angry together?

### 3.2 Theme 1: “Nobody Likes the Lockdown”

**FIGURE 2:**
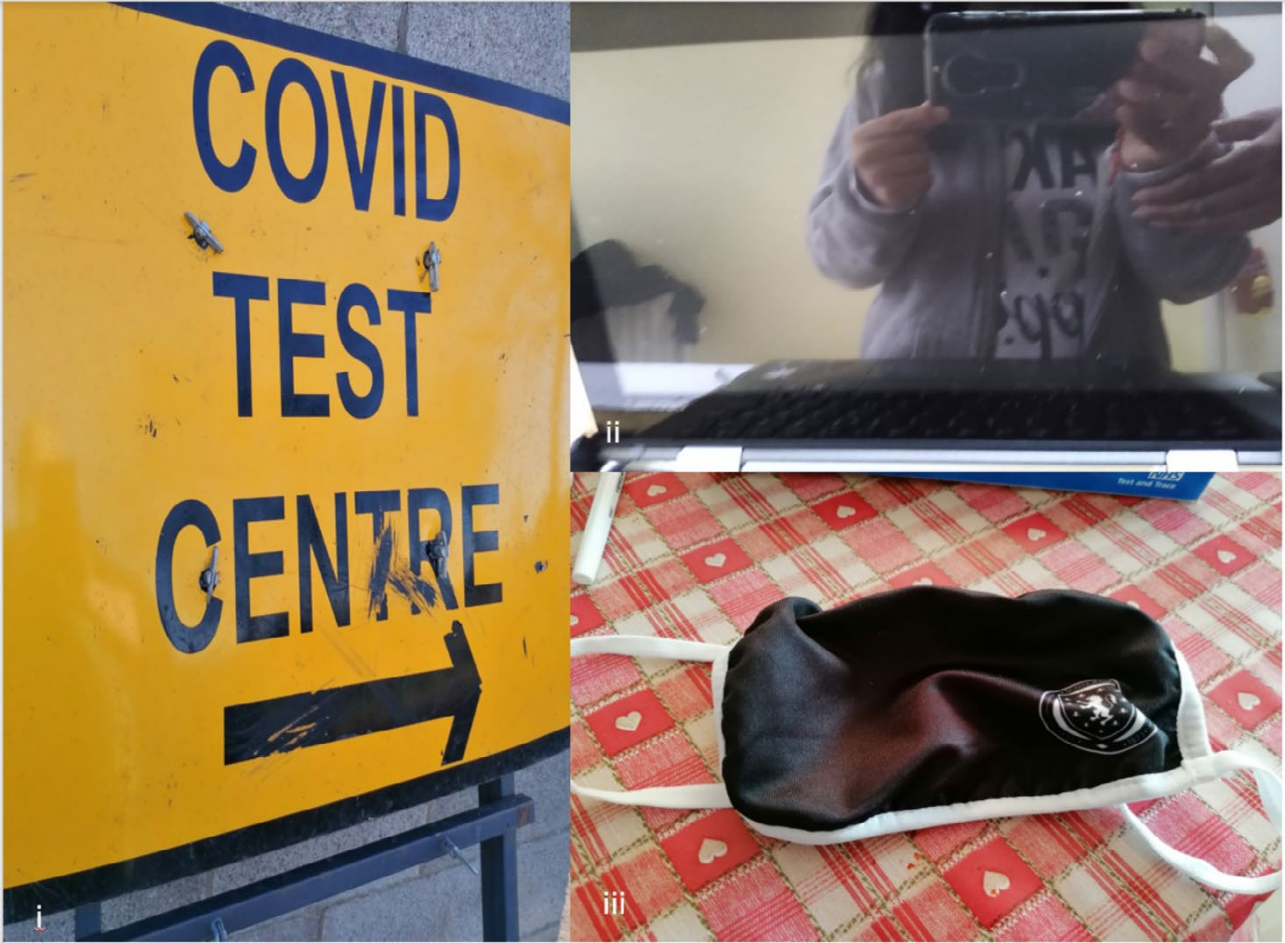
Theme 1 Images—from left clockwise (i) Covid Test Centre; (ii) Online Session; (iii) You Should Always Wear a Mask to Stay Safe.

“Nobody likes the lockdown” (see Figure 2i) describes how the children felt frustrated, sad and anxious during and after lockdown(s). This theme was characterised with ‘Stuck Inside’; ‘Covid makes me anxious’ and ‘I just want Covid to leave’. The children's negative feelings towards Covid‐19 and resulting lockdowns were often held in tension with understanding how important this was for personal and public health.

Well, I didn't like‐ I didn't like, I don't like Covid or lockdown or anything. **Meera** .

This theme centres on participants' discussions of feeling contained and missing socialising with friends and family, which they would have routinely done prior to lockdown(s). For example, **Sarah** says:

I was sad because I‐I was missing my friends.

Overall, this theme highlights the frustration and stress as a result of the pandemic and lockdown(s), and the impact this has had on both their lives during and after lockdown.

### 3.3 Theme 1a: “*Stuck Inside”*

Participants often raised notions of containment and frustration during lockdown, as they described themselves as being “*Stuck Inside*”(see Figure 2 ii). Although online meetings were often used, the children felt that these were not an adequate replacement for face‐to‐face activities.

It was‐ it was uh around Christmas time. Um, so we couldn't really go out anywhere, we had to stay in. **Maya**

This was often exemplified by the children mentioning how they missed old routines, certain family members, friends and the activities they ‘used’ to do with friends.

**Sarah**: … I was sad because I‐I was missing my friends.

Overall, “*Stuck Inside*” reflects participants experiences of stress, frustration, and anxiety in lockdown due to the sense of containment they felt.

### 3.4 Theme 1b: “Covid Makes me Anxious”

During the story telling phase during session one (which used photo‐elicitation to remind the children about Covid‐19, different lockdown rules etc. to help with retrospective recollection) the children discussed Covid‐19 and how it made them feel afraid, anxious and stressed. Seeing images of Covid‐19 and the various rules (hand washing and masks) seemed to elicit anxiety and stress. Covid was noted as being something really ‘scary’ and ‘out to get you’. As **Katie** highlights:

Sometimes I don't mind, [but] sometimes I get anxious about the lockdown.

This sentiment was reported by several participants who described Covid‐19 as though it was a monster that can cause death. This more abstract understanding of Covid‐19, coupled with the very real danger the pandemic posed, led to participants thinking about their and their loved one's mortality. Death and loss are often things children don't consider until adolescence or adulthood however Covid‐19 brought an acute awareness of mortality to the forefront.

**Ahmed**: But Covid doesn't exist.

**Researcher**: Yeah, why don't you think Covid exists?

**Ahmed**: Because I don't want to die.

Overall, ‘Covid makes me anxious’ highlights the ways in which Covid‐19 increased fear and anxiety ultimately impacting the children's wellbeing.

### 3.5 Theme 1c: “I Just Want Covid to Leave”

This theme captures how exasperated children were with Covid‐19 and the lockdown, with many talking about how they just wanted life to return to ‘normal’ again. The children would often reflect on the impact of Covid‐19 on their day‐to‐day lives and how they would feel more secure and safe in a world without Covid (Figure 2iii):

Cause’ it's been a really hard time trying to cope with using the mask all the time. And I just want Covid to leave. **Simon** .

This desire for “Covid to leave” manifested in anger for some; this anger was sometimes directed at others. For example one of the participants talked about being upset with family members for not sticking to rules and potentially causing Covid‐19 to stay around for longer. **Sarah** said ‐

Yeah I felt a little bit angry with my brother.

Overall, although the wish for Covid‐19 itself to “*leave*” was a factor, participants were generally more concerned with the change the pandemic brought. They wished for the fluctuating rules and routines of the pandemic to be replaced with ‘normal’ consistent rules and routines, allowing for a sense of permanence to return like the days before Covid‐19.

### 3.6 Theme 2: “You Don't Want the Covid [to] Get You”

**FIGURE 3:**
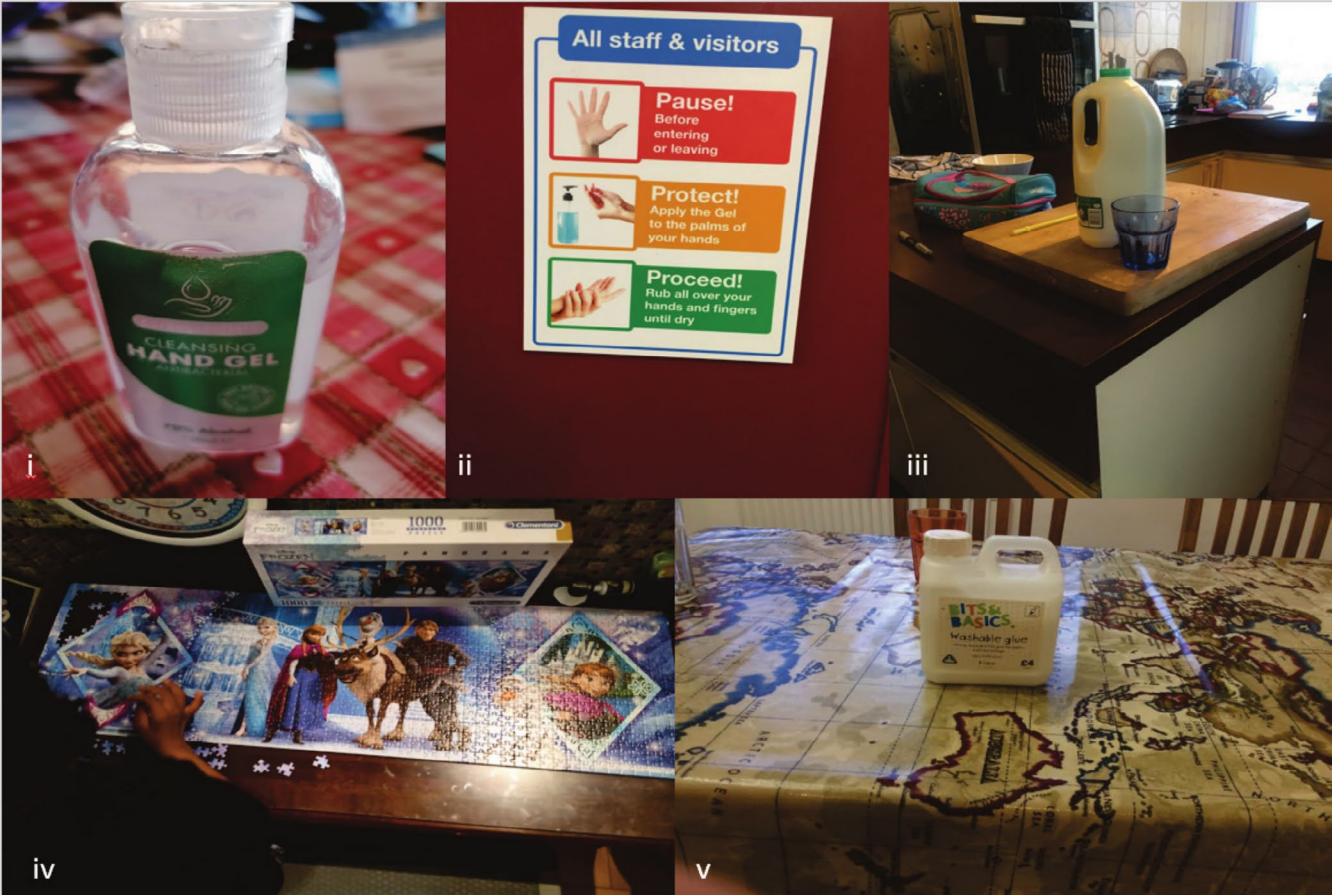
Theme 2 Images—from left clockwise (i) Hand Sanitiser; (ii) We Kill Germs; (iii) Goodnight Milk; (iv) Frozen Puzzle; (v) I peel it off.

This theme builds on the stress of the pandemic and lockdown identified in ‘Nobody likes the lockdown’, but focuses on more specific stressors and coping mechanisms employed by the participants (See Figure 3i). This theme contains three sub‐themes: ‘Obeying lockdown rules’, ‘sense of control’, and ‘It keeps me safe’. In order to combat the stress, anxiety, and fear which lockdown rules created, participants often sought to find ways in which they could feel more in control, often related to specific interests the participants have:

Yeah, well I have done it a lot. I've done it lots [of jigsaws] during the first lockdown. **Meera**

It's mostly Harry Potter Lego I'm into, really. **Simon** .

Several participants however reported more distressing cycles of rumination and associated rituals to relieve anxiety such as putting on masks all the time or reapplying hand sanitizer. Some children actively applied sanitizer whilst talking about how they cope with the pandemic:

**Rebecca:** I'm gonna put sanitizer on.

Overall, Theme 2 covers the stress and anxiety which participants attributed to the new rules and changes to everyday life imposed by lockdown(s), and how the participants coped by employing strategies that allowed them to feel a sense of safety, control and permanence.

### 3.7 Theme 2a: Obeying Lockdown Rules

This theme captures the new and increased demands commonly reported by participants including tasks such as needing to get a COVID test, isolate, wear masks, and wash hands more. Participants talked about how they would feel stressed over these new rules, discussing the need to “keep our distance” and vaccinate, with the stress of obeying these new demands often amplified by transition(s) in and out of lockdown(s). Although the children understood the reasoning behind these new rules, this didn't change the emotional impact and stress they felt (See Figure 3ii):

We're still having to wear masks too, we're still having to vaccinate when‐ when we need to. We're not‐ I mean, there's one good thing that we're able to do it, is like, to meet up with people in person, but obviously keep our distance, 5 metre distance away from them. **Simon**

The stress of obeying these new rules was also complicated due to children having to apply them and remember to apply them within different contexts. For example working out to apply rules in and during school. **Katie** says ‐

So, they got to stay two metres away, and one metre [a]way, at our school, we had to stay in our own bubble.

Overall, participants observed many new and competing demands emerging from the pandemic and the lockdown(s) they experienced. Participants felt stressed when performing them; however, they managed this stress through a multitude of ways to establish a sense of control.

### 3.8 Theme 2b: Sense of Control

The theme ‘Sense of Control’ discusses an important factor observed by the participants for navigating the new and competing demands highlighted in ‘Obeying Lockdown Rules’. This is the creation and maintenance of a sense of control and permanence during what participants observed as chaotic times. For example, getting a Covid test was generally described as “*awkward*”, with participants saying that they just “*don't like it*”, however the test was viewed more positive when a choice was provided around the type of testing offered. As highlighted by **Simon** ‐

It was kind of annoying, but the‐ there was, there was either two choices to have‐ have a bit of a test up your nose or‐ or up your throat. I did the nose one, ‘cause throat would have been a big struggle for me.

This need for control was exemplified by the importance participants put on familiarity, and routine. For example, Katie would have “*goodnight milk*” (see Figure 3iii) every night before bed, and many participants would repeatedly play the same games:

I play [Zelda on] Nintendo Switch every day. I play Nintendo Switch. **Fergus** .

Participants generally reported the use of specific interests and games as a way to maintain a sense of control. This encompassed intense interests such as, animated films, fantasy, and football, with symbols, figures, and projects related to these interests often left on display in participants homes to produce a sense of permanence (see Figure 3iv):

**Meera:** The picture is about me doing the puzzle, Frozen puzzle, because um, oh god, I don't remember! It was‐ well, it was, yeah Frozen is my favourite character. Frozen, yeah, my favourite is Frozen film. And it was in loads of pieces.

Overall, participants often sought fun and creative ways in which they could feel more in control of their lives during the pandemic, using specific interests they had to aid in this. However, it is important to note that participants also observed many more ways of maintaining a sense of comfort in control by employing repetition, routine, and compulsions.

### 3.9 Theme 2c: It Keeps Me Safe

This theme identifies the ways in which participants used control to feel safe. In this sense, it is qualitatively distinct from the previous theme ‘Sense of control’, since this was concerned with the ways in which control was used to navigate the new and increased demands associated with lockdown and the pandemic, whereas the current theme ‘It keeps me safe’ discusses the ways in which control is used to feel safe in response to the pandemic and the fear that produced:

**Researcher:** Why? Why does it [hand sanitiser] make you happy?

**Simon:** Because it makes my s‐ very happy. It makes me safe.

Several participants also highlighted the ways in which different sensory experiences kept them happy (potentially distracting them) and made them feel safe. This included the use of several different forms of sensory input, such as the use of fidget toys, hand sanitiser, and lip balm (see Figure 3v):

Umm, I like, uh I love PV glue because I put it on my hands, uh then, then I peel it off and its, I just like peeling it off [laughs]. I get, I pour, I pour, I pour some on, I pour some on my hands and then I, I, I, I, I peel, I peel it off. **Maya**.

However, many participants also observed more distressing cycles of compulsion in response to a lack of control with some children reapplying sanitizer every time the researcher mentioned Covid‐19 and their health.

**Researcher:** Are you worried about your health?

**Rebecca:** I'm gonna put [hand sanitiser on], yeah.

Overall, participants often employed different tactics to feel a greater sense of control in order to feel safe. These included the use of specific interests, and sensory experiences.

### 3.10 Theme 3: Everything Is Different Now

**FIGURE 4:**
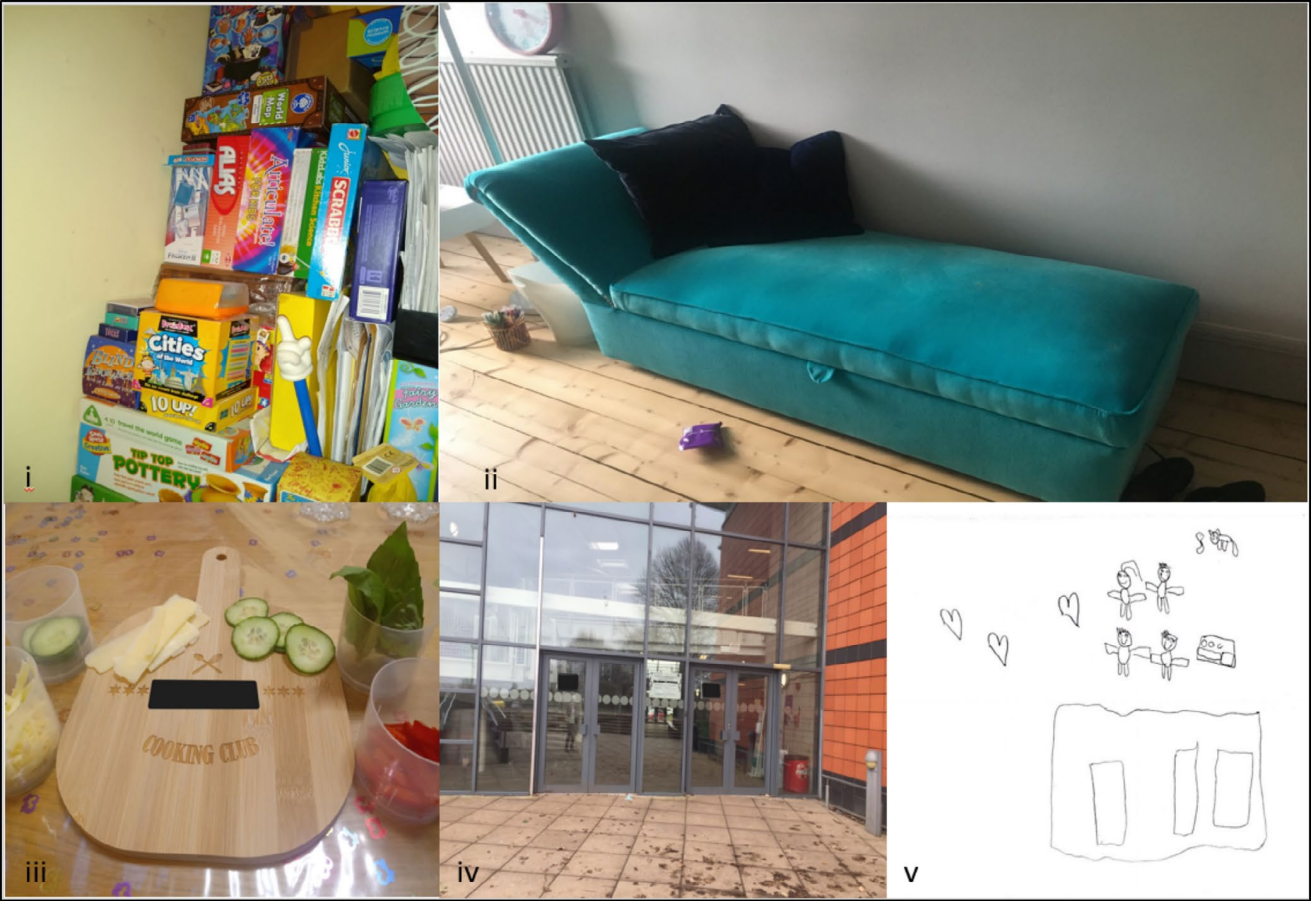
Theme 3 Images—from left clockwise (i) Games; (ii) Comfy Chatting Chair; (iii) Cooking Videos; (iv) Back to School; (v) Friends and Animals at School.

This current theme is concerned with the new normal, which participants observed during and after lockdown. This theme includes the sub‐themes ‘We stopped going’, ‘It made me happy’, ‘A new normal’ and ‘Back to School’. Reflecting on how ‘Everything is different now’, the participants highlighted the ways in which everything felt different from before, noting the new and different activities they did during the lockdown (see Figure 4i):

**Katie:** I talk to… on facetime, I talk to my grandparents.

These new activities included what made the participants happy during the lockdown(s), typically through what they perceived they had gained, such as more time with family and more time to play games:

Yeah, this pictures about the board games, and whilst‐ we play the board games during the lockdown, me and my families, and we play scrabble. **Meera**.

A key area of discussion also centred on life following the transition back into in‐person education:

**Matthew's Parent:** Did you go to school during lockdown?

**Matthew:** No.

**Matthew's Parent:** No and did you‐ would you have preferred to be at home or at school, right?

**Matthew:** School.

Overall, Theme 3 discusses the losses and gains participants noted in their day‐to‐day lives during lockdown and the transition into a ‘new normal’.

### 3.11 Theme 3a: “*We stopped going*”

This sub‐theme considers the loss of routine in and out of lockdown(s). Participants typically observed feelings of sadness and longing, as they were placed in a “*big old bubble*”, and were unable to see friends and family in person (see Figure 4ii). Although participants noted new and different ways of connecting with those in different social bubbles, these new ways were not considered adequate compared to face‐to‐face communication or in‐person visits.

We didn't see each other [a friend] in person so it was sad. **Fergus** .

**Meera:** Yeah, I really wanted to go and visit [family].

Participants also noted the loss of routines and favourite activities, as they entered into lockdown, as well as the same sense of sadness over the loss of online routines and activities (i.e., online dance classes) during the transition out of lockdown:

**Researcher**: After the lockdown, you mean, they [online dance classes] stopped?

**Meera:** Yes, they did, after the lockdown, yeah.

Lastly, the insecurity and uncertainty over the permanence of activities in and after lockdown, as well as lockdown itself, typically produced feelings of fear over the uncertainty of a future with Covid‐19:

Uhm‐well, let's just hope for the best and in the future Covid will disappear and we won't need to use masks anymore. **Simon**.

Overall, participants observed feelings of sadness and grief over the loss of in person time spent with loved ones, specific activities and routines throughout the transition in and out of lockdown(s).

### 3.12 Theme 3b: “*It made me happy*”

‘It made me happy’ follows on from the feelings of loss observed in the previous theme to discuss the perceived gains participants felt during and after lockdown(s), as they discussed the new activities they participated in as a result of lockdown (Figure 4iii):

**Meera**: Yeah, I love cooking.

**Researcher**: Yeah, yeah, and you did a lot of that during lockdown.

**Meera**: Yeah, I did.

This included a range of creative activities that participants were offered throughout lockdown, such as art, cooking, and dancing as well as participants highlighting the joy they found in being able to spend more time with their family and doing different activities with them:

I love doing online dancing during lockdown. **Meera**.

**Maya:** I like to play with the bat.

Moreover, participants also highlighted the various activities they enjoyed during lockdown which were organised by external groups and charities.

**Researcher:** So, you like to take videos [during lockdown]?

**Meera:** Yeah, well, sometimes for association. [meaning Down Syndrome association].

Lastly, participants discussed the joy they found in having more time to play both board and video games throughout lockdown:

**Researcher:** What was your favourite thing to do during the lockdown?

**Simon:** Well, I like playing my Xbox when I'm back home.

Overall, participants typically found the new activities offered during lockdown made them happy; however, the key aspect which appeared to bring the most joy to participants during lockdown was simply being able to spend more time with family and having more time for their favourite activities.

### 3.13 Theme 3c: A New Normal

‘A new normal’ focuses on participants’ thoughts, feelings, and understanding of day‐to‐day life during the lockdown transition phases. The children reflected on what they did and how they felt before, during, and after Covid‐19, with some behaviours or cognitions remaining, such as wearing masks, using sanitizer, and ‘being careful’.

**Researcher:** Do you feel like your life is back to normal?

**Katie:** Yes.

**Researcher:** Yeah, you weren't able to visit, and how do you feel are‐ are you still‐ are you visiting family now or?

**Simon:** Sometimes.

**Meera:** Yeah, I do visiting my families.

**Researcher:** Yeah, yeah, sometimes so you still‐ I mean are you still like, being careful or are you, you can just go?

**Simon:** Yeah, still being careful.

**Meera:** Yeah, I am very careful.

This sense of a new normal was typically orientated towards two different observations; first, participants highlighted their excitement to do the things they missed during lockdown again:

I like to go to college now. **Rebecca**.

**Matthew:** Yeah, I go there [my grandparents' house] on Monday. To watch movie. Marvel film.

Second, participants also discussed the activities and connections they lost in the transition out of lockdown:

**Researcher**: And are you now, still‐ are you still dancing now?

**Maya:** No. Maybe we did them for little bit, maybe 2 sessions.

**Researcher**: OK, but like the online sessions, they've stopped now?

**Maya:** Yes, they have stopped ‘cause of Covid lockdown happening.

Whilst participants were keen to play games with friends and meet with relatives again, they also observed sadness over the loss of activities and changing relationships as they transitioned out of lockdown:

**Researcher:** Yeah. Were you sad to‐to leave your brothers and sisters and go back to school?

**Katie:** Yeah.

Overall, participants discussed a new normal they felt they entered in the transitions in and out of lockdown, the lack of certainty and permanence these transitions seemed to bring, and their excitement for a return to a perceived sense of normality whilst also grieving the loss of connections and activities made during lockdown.

### 3.14 Theme 3d: Back to School

This theme captures a specific aspect of the new normal identified in the previous theme by considering the transition back into face‐to‐face education following lockdown (see Figure 4iv). When participants were asked to draw a picture (during session one) they would often draw scenes about their journeys to school or being at school (e.g., sitting at their desk next to friends) indicating that school was important to them.

Yeah, more than online yeah, because we go – we actually go‐ we physically going to school and… and I like it, and I like it. **Meera** .

It was very, it was very hard. I just rather, I just rather went to college, but because, because we had lockdown, so it wasn't, it wasn't so easy. **Maya** .

Participants were typically excited to return to school and longed for that face‐to‐face connection again, including with animals that some participants could see at their school (Figure 4v):

**Researcher**: And do you think, um, like, face to face lessons are more fun?

**Katie:** Oh yeah.

Several participants discussed the difficulties they observed in the transition back into school. The difficulty in returning to school was captured over four aspects: lack of familiarity, anxiety about returning, needing to wear a school uniform again, and sadness over no longer seeing family during the daytime. However, the main difficulty, as Katie succinctly summed up, was that they were just not, “*used to it*”:

The first day back to school was a bit… tricky… I wasn't used to it. **Katie** .

Nevertheless, participants typically highlighted how much they enjoy school and seeing their friends again:

[Being in] school is much easier to study. **Hannah** .

Moreover, doing school face‐to‐face again was a welcomed relief for many of the participants due to the difficulty and boredom they noted around online schooling compared with the enjoyment they felt about being in‐person:

Sometimes difficult [doing school online]. **Matthew** .

Overall, participants observed a longing to return to in‐person education, yet typically found difficulty in the transition as they felt they were no longer “*used to it*”; nevertheless, the participants often highlighted their enjoyment in returning to in person schooling and seeing their friends again.

## 4 Discussion

The research question was ‘What is your experience of Covid‐19 and the Covid‐19 lockdown(s)?’ The themes generated included the overarching theme of (Im)Permanace, with themes including: “Nobody likes the lockdown”; “You don't want the Covid [to] get you”; and ‘Everything is different now’. The overarching theme of (Im)Permanace highlights the stress and anxiety the children experienced as a result of needing routine and structure during the turbulent and unpredictable time of Covid‐19 lockdown. This tension between routine and change was felt by the children and seemed to play a role across all of the other related themes. This need for routine to help improve regulation and reduce anxiety in children with intellectual disabilities has been reported by other stakeholder groups such as teachers (Girgis et al. 2024). Parents also highlight that routines help support children with intellectual disabilities as well as provide family cohesion and functioning (Heiman 2002) indicating that routine is important not only for the children themselves but also the wider family. The ever‐changing routine and rules also led to palpable uncertainty about the future which the children in the current study reported, similar to research also documenting uncertainty for the future in adults with Intellectual Disabilities (Carey et al. 2021).

The theme ‘Nobody likes the lockdown’ included subthemes around being stuck inside for socialising and school work. The children explained that they wanted to go back to school and not have to do online learning. This is again in line with what parents reported about home learning especially if the children's needs were different at school compared to the home environment (Averett 2021). The children also talked about socialising online and how the only way to see their friends was through facetime apps and online gaming platforms (like Animal Crossing). Previous research in neurotypical children highlighted the crucial role that school plays not only in children's learning but also children's socialising (Larivière‐Bastien et al. 2022). Therefore the loss of school not only had an impact on their academic motivation, and learning but also on their socialising abilities and opportunities. Despite Covid‐19 lockdown being reported as an opportunity to potentially galvanise improved social inclusion for children with intellectual disabilities (Beaton et al. 2021) the children in the current study said that they preferred seeing friends in person. Additionally, the children talked about being so scared of Covid‐19 that they likened it to being a ‘monster’ or not existing at all. This use of imagination as a method of meaning‐making is a way to make sense of the strange new environment the children found themselves in (Brockmeier 2009). Related to this was the realisation of the precarious nature of mortality—a concept that is often conceptualised as a remote possibility in middle childhood years and is not something defined with finality and universality until pre‐adolescence (White et al. 1978). In addition to this it is reported that children who have lower academic abilities show delayed comprehension of this finality (Hopkins 2014). Children (Pompele et al. 2022) and adults with Intellectual Disabilities (Lake et al. 2021) reported an increased fear of death indicating that anxiety around bereavement or one's own death was a common fear that gripped many vulnerable groups including the current group of children with Intellectual Disabilities.

In the theme ‘You don't want the Covid to get you’, the children talked about coping strategies they would employ to deal with the anxiety and also the boredom associated with lockdown. The coping strategies adopted were similar to adults with Intellectual disabilities and neurotypical children with the current participants reporting that they took up dancing, art, cooking (Montreuil et al. 2023) as well as meeting with friends and family virtually to reduce feelings of loneliness and isolation (Lake et al. 2021). What may be distinct in the current group was the role of special or intense interests such as sensory stimulations, computer games and jigsaws. Despite intense interests being well documented in Autistic children (e.g., Anthony et al. 2013; Murray 2018; sometimes referred to as monotropism, Wood 2019), little is reported around the special or intense interests of children with intellectual disabilities except earlier publications around such interests being used to facilitate learning (e.g., Hearne and Stone 1995). Some children in the current study talked about doing sensory based tasks that they enjoy such as applying glue to their hands and then picking it off which may have been used to reduce anxiety or improve mood. In a similar vein the children also mentioned adhering to the lockdown rules via sensory based activities such as applying hand sanitiser, washing hands and wearing masks, which allowed them to feel a certain sense of control.

Moreover, the children also offered a positive view of Covid‐19 with the theme ‘The new normal’ where they mentioned how they enjoyed spending time with family, playing games, and doing creative activities together such as cooking. The current study therefore shows that despite high levels of anxiety, the children had also experienced some positives with more time being spent with the family and enjoying the accessible ways to do activities (e.g., online cooking and dance classes). What was distinct about the current study was that the children with Intellectual Disabilities didn't report family conflict which was noted by child groups (Montreuil et al. 2023) and adults with Intellectual Disabilities (Kim et al. 2021). This may be caused by difficulties in social competence and understanding complex dynamics (such as conflict resolution) reported in children with Intellectual Disabilities (e.g., Wieland et al. 2014). Therefore, conflicts may have taken place but were perhaps not recognised or understood by the current participants. Further research should include children and their parents to better understand whether conflicts took place in the home during Covid‐19 and how the family mitigated these.

Additionally, there were still fears noted by the children regarding this transition with many reporting that they feel different about going back to school. Children with intellectual disabilities struggle with transitions and often show increased levels of aggressive outbursts or self‐injurious behaviours if communication breaks down (Castillo et al. 2018), therefore, further research is needed to explore how the children and their families were impacted by transitioning back to school and whether further support is needed to reduce this distress. Despite having worries about transitioning back to school, the children also talked about missing school and missing their friends or activities that they did at school—this was evidenced during session one where they were asked to draw a picture. Most of the children drew scenes in the classroom sitting at their desk with friends, travelling to school with friends, or the animals they would see during their journeys to school. This shows the important role school plays in providing opportunities for the children to socialise and connect, which was similarly reported by neurotypical children (Larivière‐Bastien et al. 2022).

The current study is the first study that has captured the experience of Covid‐19 lockdown in children with intellectual disabilities themselves; however, there are some limitations that must be acknowledged. At the time of the study, some of the children had returned to school and some were still transitioning back; therefore, there was a mixture of online photovoice sessions and in‐person sessions (at a school). As such, some of the group members knew each other and some did not. This could have impacted the group dynamics and influenced how open the children were about their experiences. It is worth also noting that although Photovoice is viewed as an inclusive method (Booth and Booth 2003; Rose 2014; Williamson et al. 2020), it still requires a level of understanding regarding the content of the image and what the image represents. Therefore, this method cannot be used by those who have severe and profound intellectual disabilities. In line with this, some children could do the photovoice sessions themselves; however, others needed a parent to support them. This could have impacted on what the child said and what the child chose to focus on; however, it was judged that it was more important to ensure sessions were accessible for any child who wanted to take part, including those who needed additional support.

In conclusion, the current study explored what life was like for children with intellectual disabilities during the Covid‐19 pandemic. The themes identified included the overarching theme of (Im)Permanace, with themes including ‘Nobody likes the lockdown’, ‘You don't want the Covid to get you’, and ‘Everything is different now’. The children explained the difficulties trying to maintain routine and order during an unpredictable time. They highlighted the anxiety they felt and continue to feel even though ‘normality’ was slowly returning. This shows that despite lockdown lifting the children still remained quite anxious and this should be taken into consideration for any children having difficulties transitioning back to school and taking part in face‐to‐face activities. The children also mentioned coping strategies they employed such as enjoying sensory‐based activities or interest‐based activities which distracted them and gave them a sense of control over their immediate environment. Further exploration of mental health in children with intellectual disabilities as well as the coping strategies they employ is needed. Ripple effects of the lockdown may continue to be an issue for these children and more support may be needed for many years to come.

## Funding

This work was supported by Economic and Social Research Council, ES/W001985/1.

## Ethics Statement

Ethics was approved for this study from the University of Edinburgh, Clinical Psychology Ethics Committee approval—CLPS099.

## Consent

Appropriate permissions and consents were obtained before data was collected.

## Conflicts of Interest

The authors declare no conflicts of interest.

## Supporting information


**Data S1:** Supporting Information.

## Data Availability

Data currently available on request from the authors; however, it will be openly available after 31st August 2024 on Edinburgh DataShare https://datashare.ed.ac.uk/.

## Endnotes

^1^
Although it is acknowledged that *emotion regulation* and *coping* demonstrate conceptual overlap, these concepts have distinct targets, notably *emotion regulation* targets emotion and *coping* targets external stressors (Trudel‐Fitzgerald et al. 2024, 3). The current study (which is interested in the impact of an external stressor—Covid‐19 pandemic) therefore focuses on *coping*.
