# Supplementary material for: ‘Let's Just Hope for the Best’: A Photovoice Study Exploring the Impact of Covid‐19 Restrictions on Children With Intellectual Disabilities in the UK
Source: J Appl Res Intellect Disabil. 2026 Aug 28;39(5):e70298. doi: 10.1111/jar.70298 (PMC13525080; doi:10.1111/jar.70298)
Supplement: Supplementary file 1 [file JAR-39-e70298-s001.docx]

**Supplementary Material – Full study protocol**

Road to Recovery Research Project:

Interview and Photovoice protocol

Version 6.0 - Last update: 4 February 2022

# 1. Study sample

A. **Who is the study sample?**

- 20 children aged 5 to 17 with mild to moderate intellectual disability (ID) residing in the UK (10 children in the primary-school age 5 to 11 years, and 10 children in the secondary school age 12 to 17 years). These children will be participants in group sessions.
- 20 adult caregivers/parents: 10 parents of children who have mild-moderate ID (these will be the direct parents or caregivers of the child participants - 1 caregiver per participant), & 10 parents of children with severe to profound ID. Parents will be interview respondents.

B. **What are the eligibility criteria?**
a) Children:

The table below summarizes the main eligibility and exclusion criteria for children in the group sessions:

Eligibility criteria for children with ID in the group sessions

Children aged 5-17 with mild-moderate intellectual disability residing in the UK

Both males and females

Various educational status: currently in education - not in education - special education - mainstream education

Various socio-economic status

Ability to understand simple “information-carrying words” in the English language

Note: ID can be associated with other disabilities (i.e. physical, neurodevelopmental, etc.)

Exclusion criteria for children with ID in the group sessions

Other disabilities without ID

Severe to profound ID

b) Parents/caregivers:

The parents/caregivers of 10 out of the 20 children from the group sessions will be interviewed individually. Additionally, 10 parents of children with severe to profound ID will be identified and invited to an interview.

C. **What is an intellectual disability (ID) and what are the levels of ID?**

- ***Intellectual disability*** is characterized by “significant limitations both in intellectual functioning and in adaptive behavior as expressed in conceptual, social and practical adaptive skills. This disability originates before age 18”, according to the WHO International Classification of Diseases.(World Health Organization, 2019)
- ***Mild intellectual disability:*** “Affected persons often exhibit difficulties in the acquisition and comprehension of complex language concepts and academic skills. Most master basic self-care, domestic, and practical activities. Persons affected by a mild disorder of intellectual development can generally achieve relatively independent living and employment as adults but may require appropriate support.”(World Health Organization, 2019)
- ***Moderate intellectual disability:*** “Language and capacity for acquisition of academic skills of persons affected by a moderate disorder of intellectual development vary but are generally limited to basic skills. Some may master basic self-care, domestic, and practical activities. Most affected persons require considerable and consistent support in order to achieve independent living and employment as adults”.(World Health Organization, 2019)
- As opposed to mild-moderate ID***,*** *persons affected by* ***severe to profound intellectual disability***  “exhibit very limited language and capacity for acquisition of academic skills. They may also have motor impairments and typically require daily support in a supervised environment for adequate care”.(World Health Organization, 2019)

Severe to profound ID is included in the exclusion criteria for participation in the children group sessions, since participatory research involving individuals with severe-profound ID requires a different scope and different tools. However, given that it is very important to capture the experiences of children with severe to profound ID and their families throughout the pandemic, transition and recovery phases, interviews with 10 parents/caregivers of children identified as having severe to profound Id will be carried out.

**D. How will eligibility be assessed?**

- While quantitative IQ (intelligence quotient) tests can be considered as the most standard form of assessment of intellectual disability, no formal standardized IQ tests will be conducted with participants in this study for the following 2 reasons:

1. The approach of the study strives to be emancipatory and participatory, focusing on the agential role of individuals with ID, as opposed to other models such as the medical model of disability which presents disability as a medical concern linked to abnormality of functioning. In this respect, putting a statistical number to the individual’s level of intellectual functioning seems restrictive and opposes the overall approach of the study.
2. There is an assumption that the organizations/institutions providing services for children with ID would have already assessed their beneficiaries. The form of assessment used will be inquired about through conversations with the administrators, gatekeepers and key informants.
3. The appropriateness of standard IQ tests may be debatable for use with certain groups of children, especially those with severe to profound intellectual disability.

- Alternatively, the level of intellectual disability will be determined *qualitatively* through formal and informal discussions with the organization gatekeeper during the recruitment phase, as well as through individual and family accounts, with the assistance of disability experts who will have an advisory and co-facilitation role throughout data collection. These discussions will have 2 main objectives:

1) to ensure that the respondent is eligible and does not have any of the exclusion criteria (e.g. severe or profound ID);

2) to determine if the person has the capacity to provide informed consent. In fact, and according to the IASSIDD Ethics Guidelines for International, Multicenter Research Involving People with Intellectual Disabilities ((Dalton and McVilly, 2004b)), the researcher should be able to verify the following:

- “the form and extent of [the respondent’s] decision-making capacity in everyday situations;
- their propensity to acquiesce and/or demonstrated ability to say “no” in circumstances not to their liking;
- their ability to interpret and explain in their own words the protocol that has been presented to them, including their appreciation of any risks and their right to decline participation without any adverse consequences to them or the services they receive [and excluding technical information]”(Dalton and McVilly, 2004b) p.62. This will be inquired about through the discussions with the children, their parents/caregivers and the gatekeepers, as well as through the information session with children.
- The levels of communication and comprehension of the respondents, and whether the respondent has the ability to understand simple “information-carrying words”: will be assessed through one-to-one activities that aim to qualitatively evaluate word logic, reading level, memory and attention, as well as the respondent’s learning style. These will be developed and facilitated by the disability expert. The intent of this assessment is not discriminatory, but has 2 objectives:

1) to ensure that the respondent has the ability to interpret and explain in his or her own words the consent form;
2) to serve as a guide to make reasonable adjustments to the subsequent data collection process, by making sure to adapt the communication style and tools to the person’s comprehension and communication levels (e.g. using more pictures and less text, or vice versa, etc.), as well as using the appropriate augmentative and alternative communication techniques, for every single respondent based on their personal preferences and abilities.

- The child’s previous experience with online learning, as well as access and ease to use online interactive platforms (such as Zoom, Skype, Microsoft Teams, etc.) will also be inquired about, to ensure their meaningful participation in the group sessions if they were to be carried out online.

# 2. Selection of participants

Selection of study participants will be based on purposeful and convenient sampling methods. Data collection will include face-to-face and/or online methods (see section on Methodology). The following figure illustrates the steps in the recruitment process of study participants:

1. **Site selection based on pre-defined criteria:** Organizations/charities and schools working with children with ID will be selected based on relevance to the research and on the following criteria:
a. Charity organizations providing leisure, cultural or edutainment activities for children and adolescents with disabilities in the UK (England & Scotland)
b. Schools/institutions (special education or mainstream) in the UK (England and Scotland)
c. Self-advocacy groups for children and adolescents with intellectual disability
d. Based in the UK and/or serving UK-bound communities (England & Scotland)
2. **Contacting site administrator and explaining purpose of research:** After approval, representatives from these organizations/institutions will act as gatekeepers for outreach to children with intellectual disability and their parents/caregivers. The administrator helps identify the assigned gatekeeper within each organization/institution.
3. **Identification of gatekeeper:** The gatekeeper is a staff member of the organization/institution who will be appointed by the administrator or the manager to serve as a coordinator or middle person for outreach between children with ID, their parents and the research team. An information package will be shared with the gatekeeper, and the researcher explains the purpose and scope of the research to the appointed coordinator in each organization/institution, as well as the eligibility and exclusion criteria.
3. **Gatekeeper identifies, and contacts potential participants’ parents/guardians based on eligibility criteria, for preliminary consent, after discussion with researcher:** The gatekeeper develops a list of potential study participants (children) within the organization for the study based on the eligibility criteria (see “Eligibility Criteria” in section 1B). Preliminary “approval” or “interest” for children to participate will be sought through the gatekeepers, who will also ask parents/caregivers if their details (name, phone number, personal information about the child such as age, communication level, previous experience in online learning, access to online platforms, etc.) can be shared with the researcher. The gatekeeper also makes it clear to the respondent that their decision to participate in the study or not, will not affect their relationship with the gatekeeper organization nor affect any kind of services they are already receiving from the organization. The consent process will go through the parents or caregivers before approaching children with ID. This approval does not qualify as an official consent, but constitutes a preliminary expression of interest and an ethical requirement to make sure the researcher can have access to the contact list.
4. **Gatekeeper shares contact information with the researcher:** The researcher, supported by the disability experts, reviews the contact list and information and selects a preliminary sample from the list to be approached for recruitment and consent (and also selects replacements), after making sure the potential candidates correspond to the eligibility criteria, and which provides a variety of educational/socio-economic backgrounds. The researcher also gathers informal information about the potential participants, especially concerning the child’s communication levels and ability to give assent.
5. **Trust-building through observations of activities or participation in informal events within selected organizations/institutions and informal discussions with potential participants (children with ID and their parents):** this would be considered as the first step towards recruitment of study participants. It aims to adopt a personal approach (Nicholson et al., 2013) with a more prolonged engagement (Sigstad and Garrels, 2017), which has been encouraged in similar studies highlighting the advantage of the researcher being informally introduced to potential participants with ID in building trust and ensuring better informed consent (Sigstad and Garrels, 2017) (Nicholson et al., 2013) (Carey and Griffiths, 2017).
7. **Recruitment of caregivers/parents for interviews**: Parents/caregivers will be contacted by phone to gather informed consent and set interview time and date. For parents of children with mild-moderate ID, consent for their child to participate in the group sessions will also be asked, including specific consent considerations related to using photovoice and other photographic methods (see note below). Participant information sheet details and written consent forms will be presented at the beginning of the online or face-to-face semi-structured interviews. The researcher also makes it clear to the respondent that their decision to participate in the study or not, will not affect their relationship with the gatekeeper organization nor affect any kind of services they are already receiving through the organization.

***Important note on consent for using photographic images:***

Parental consent and child assent will include 3 levels of consent in relation to the use of photographic methods:

a) Consent to participate in the group sessions which include photographic methods
b) Consent to be photographed and/or appear in photographs taken during the group sessions for research purposes
c) Consent to the use of any photograph taken during data collection for publication and/or public dissemination (consent will be acquired for each individual photograph)

Specific instructions will be provided to the children in the camera training workshop (Session 3) related to what can and cannot be photographed (e.g. public vs. private spaces, the inside of schools and/or specific identifiable persons cannot be photographed in a school setting). It is also noteworthy to mention that verbal consent from non-participant individuals being photographed will need to be acquired prior to having their photo taken if their faces are to be shown in the photos.

Information about the storage and management of the digital photographs taken will also be provided to the parents and children in the participant information sheet. Digital photos will be shared via a secure photo-storage application, which is encrypted and password-protected. Identifiable IP addresses will be deleted.

8. **Recruitment of children for group discussions** : After parental consent, the identified children with mild-moderate ID will be divided into 4 groups based on their age and country of residence: two groups for primary-school age children aged 5 to 11 (England & Scotland), and two groups for secondary-school age children aged 12 to 17 (England & Scotland). The target group number is 5 children per age group, to allow meaningful participation and favorable group dynamics. Children group sessions will be carried out either face-to-face or online (see section on methodology), based on feasibility and continuous health & safety assessments.

- For face-to-face sessions: children will primarily be recruited through organizations/charities and may already be part of pre-existing group (e.g. from playschemes, weekly activities, etc.)
- For online sessions: children primarily recruited through schools/institutions, and it is recommended not to have more than 3-5 children in the same online session to allow good participation.

9. **Introductory session for assent with children with ID (for children whose parents/caregivers have provided consent for them to participate in the study).** An introductory session will be organized to acquire children assent, before the group sessions begin. The session can either be individual or group-based.

This first session is an introductory session and aims to explain the purpose of the research, and ensure children are well informed before providing their assent to participate. The session will be co-facilitated by disability expert and/or speech therapist, to ensure optimal communication. It should present itself as a non-threatening session, which could be carried out with the presence of the coordinator depending on the participant’s comfort level. It will include an ice-breaker, an introduction and a trust building activity. Details of the research will then be presented and a visual participant information sheet is handed to the participant (Cf. Child assent form). This will also include a photo of the researcher and concrete information about them, so that the participant can always identify the research team (Nicholson et al., 2013). Through conversation, the researcher then verifies if the participant understood the details of the research before asking for consent. The researcher’s role should be to make sure the respondents understand the risks of research and voluntarily consent (Iacono, 2006). If the participant is deemed capable of providing consent and shows understanding of the research, the researcher asks for oral assent. The gatekeeper acts as a witness to the consent, as stipulated in Ethics Protocol. If the participant provides assent, then an assessment of their communication and comprehension levels is conducted, followed by the group sessions. Assent can either be requested during the introductory meeting or at a later meeting if the participant wishes to have more time for reflection. The key is to ensure *sufficient accessible information* and *sufficient time for decision-making* (Carey and Griffiths, 2017, Northway et al., 2015). The researcher also makes it clear to the respondent that their decision to participate in the study or not, will not affect their relationship with the gatekeeper organization nor affect any kind of services they are already receiving from the organization.

Children will be identified via the organization’s gatekeepers (e.g., teachers or charity staff) who will know the child’s level of functioning and if they would have the ability to participate in the current study. If possible we will also invite these gatekeepers to peripherally assist with the groups.

**10. Group sessions with children (whose parents/caregivers have provided consent for them to participate in the study):** these will be carried out using various photographic and visual methods. A minimum of 5 sessions per group will be conducted, either online or face-to-face. (See section on methodology for further details)

11. **Continuous feedback – ongoing consent with study participants and coordinators: There will be a “constant vigilance of signs of assent or dissent” (Carey and Griffiths, 2017), especially** at each new phase of the research, which is considered as “process consent” (Dalton and McVilly, 2004a). This is recommended by the IASSIDD Ethics Guidelines 2004, in order to ensure that the participant fully understands and maintains consent to participate throughout the research (Dalton and McVilly, 2004a).
6. .

# 3. Methodology

- **Parents/caregiver interviews:** 20 semi-structured interviews (1 hour each), including 10 interviews with a parent/caregiver of a child with mild-moderate ID, and 10 interviews with a parent/caregiver of a child with severe-profound ID.
- **Children group sessions:** 20 children aged 5 to 17 with mild-moderate ID, divided into 4 groups:
  - 5 children aged 5 to 11 based in England
  - 5 children aged 5 to 11 based in Scotland
  - 5 children aged 12 to 17 based in England
  - 5 children aged 12 to 17 based in Scotland

A total of 5 sessions (60 to 90min each) will be carried out per group. Methods include using various photographic and visual material such as photovoice, photo-elicitation, pictorial mapping, and visual storytelling. Methods will be adapted based on the age group and children’s preferred methods of communication which will allow meaningful participation. The content of the sessions can be broken down as follows:

1) Session 1: Introductory session for assent and trust-building
2) Session 2: Storytelling *(to gather retrospective information)*
3) Session 3: Camera training workshop + 1^st^ photography assignment
4) Session 4: Discussion on the 1^st^ assignment + 2^nd^ photography assignment
5) Session 5: Discussion on all photographs + wrap up

**Online adaptations to the photovoice protocol**

Given the COVID-19 pandemic and the emergence of new variants which necessitate further restrictions, carrying out face-to-face may not always be feasible, especially with children and youth with disabilities. Adaptations can be made to the original photovoice protocol when shifting to online data collection and using an online videoconference platform such as Microsoft Teams. Such adaptations are important to ensure the meaningful participation of the children and youth with ID, while acknowledging some of the methodological limitations in using online platforms.

These adaptations can include but are not limited to the following:

- **Recruitment**:
  - Designing a ‘recruitment poster’ and developing an online sign-up sheet (e.g.Google form format) which can both be disseminated through charities, schools and online social media platforms to reach a maximum number of potential participants.
  - Given the qualitative nature of the method, convenience and/or snowball sampling techniques may also be used to promote sign-up.
  - Restrictions of country-specific groups may be removed, and mixed country-groups can be formed (i.e. Scotland and England), given online accessibility and reach.

- **Session design**:
  - Reducing the number of sessions to 3 instead of 5, given the difficulty in ensuring sustained attendance online across consecutive weeks, with participants often having conflicting time schedules and time availabilities. A suggested breakdown of activities can be as follows:
    1) Session 1: Introductory session for assent and trust-building, then drawing and storytelling activities (after consent)
    2) Session 2: Camera training workshop + mini-photography exercise during the session + photography assignment for session 3
    3) Session 3: Discussion on photographs + wrap up
  - Reducing the number of participants to 3 to 5 maximum per group (per session), to ensure that every participant has time and space to actively participate and share with others. This is also important to allow space for an online interaction between the participants.
- **Tools**:
  - Modifying all tools to a clear visual format to be used online.
  - Using ice-breaking and trust-building activities to briefly assess each participant’s ability to interact and communicate online
  - Maximizing the use of online technology to share photographs and drawings (i.e. Microsoft PowerPoint as a visual tool).
  - Carrying out a mini-photography exercise during the session, where participants are asked to take photographs within their home (15 min assignment), and discuss the photographs afterwards, so that the facilitator can both assess the level of engagement of participants in the activity and give feedback on photography principles.

# **4. DATA MANAGEMENT**

### **Personal Data**

### The following personal data will be collected as part of the research:

- Name
- Email address

### Personal data will be stored by the research team at The University of Edinburgh in a password protected folder on the University’s secure VPN. Only core members of the research team (the Principal investigator and study researchers) will have access to personal data.

Personal data will be stored for up to 5 years.

### **Transfer of Data**

Data collected or generated by the study (including personal data) will not be transferred to any external individuals or organisations outside of the Sponsoring organisation(s).

### **Data Controller**

A data controller is an organisation that determines the purposes for which, and the manner in which, any personal data are processed.

The University of Edinburgh is the data controller along with any other entities involved in delivering the study that may be a data controller in accordance with applicable laws (e.g. the site)

### **Data Breaches**

Any data breaches will be reported to the University of Edinburgh Data Protection Officer who will onward report to the relevant authority according to the appropriate timelines if required.

### **Data Information Flow**

Consent obtained

Demographic information collected (inc. name, contact details, household composition, employment status, martial statis, sexuality, race. Plus, child DOB, sex, and diagnosis(es) if known)

Interview data collected will all be anonymised. Participant (parent) name replaced with a digital identifier and any identifiable information provided will be omitted from the transcript

Child photovoice data collected: child’s pictures that they have taken, audio and video recordings the group discussions during the photovoice activities

Any photos containing identifying information will be destroyed.

Following transcription, all audio recordings will also be destroyed.

All data being kept will be labelled using the participant’s digital identifier.
All data will be stored on the University’s VPN. Data will be stored for up to 5 years.

**References cited**

CAREY, E. & GRIFFITHS, C. 2017. Recruitment and consent of adults with intellectual disabilities in a classic grounded theory research study: ethical and methodological considerations. *Disability & Society,* 32**,** 193-212.

DALTON, A. J. & MCVILLY, K. R. 2004a. Ethics Guidelines for International, Multicenter Research Involving People with Intellectual Disabilities. *Journal of Policy & Practice in Intellectual Disabilities,* 1**,** 57-70.

DALTON, A. J. & MCVILLY, K. R. 2004b. Ethics Guidelines for International, Multicenter Research Involving People with Intellectual Disabilities. *Journal of Policy and Practice in Intellectual Disabilities,* 1**,** 57-70.

IACONO, T. 2006. Ethical challenges and complexities of including people with intellectual disability as participants in research. *Journal of Intellectual and Developmental Disability,* 31**,** 173-179.

NICHOLSON, L., COLYER, M. & COOPER, S. A. 2013. Recruitment to intellectual disability research: a qualitative study. *Journal of Intellectual Disability Research,* 57**,** 647-656.

NORTHWAY, R., HOWARTH, J. & EVANS, L. 2015. Participatory research, people with intellectual disabilities and ethical approval: making reasonable adjustments to enable participation. *Journal of Clinical Nursing,* 24**,** 573-581.

SIGSTAD, H. M. H. T. & GARRELS, V. 2017. Facilitating qualitative research interviews for respondents with intellectual disability. *European Journal of Special Needs Education***,** 1-15.

WORLD HEALTH ORGANIZATION 2019. 6A00 Disorders of intellectual development. *International Classification of Diseases 11th Revision.*
